# Interpreting molecular evidence for biosecurity decision-making: Lessons from coconut rhinoceros beetle incursions in the Pacific

**DOI:** 10.1016/j.cris.2026.100131

**Published:** 2026-07-21

**Authors:** Kayvan Etebari, Michael J. Furlong

**Affiliations:** a School of Agriculture and Food Sustainability, The University of Queensland, Brisbane, Australia; b School of the Environment, The University of Queensland, Brisbane, Australia

**Keywords:** *Oryctes rhinoceros*, Biosecurity diagnostics, Host-pathogen interactions, Nudivirus, Haplotype, Invasive species, Molecular tools, Genomics

## Abstract

Molecular and genomic tools are increasingly used in biosecurity to support early detection, diagnostics and reconstruction of invasion pathways. However, selecting an appropriate marker for the question being addressed is critical, and translating molecular evidence into operational decisions can be challenging. Over-interpretation of commonly used markers may lead to misleading conclusions about population structure, invasion history and management priorities. DNA barcoding remains valuable for species identification and initial haplotype assignment, but questions concerning invasion pathways, admixture and phenotypic variation require additional molecular and ecological evidence. Here, we use coconut rhinoceros beetle (*Oryctes rhinoceros*) incursions in the Pacific as a case study to highlight interpretive pitfalls and lessons for applying molecular and genomic tools in biosecurity and biological control. We synthesise evidence on the strengths and limitations of mitochondrial markers, particularly *CoxI*, for haplotyping and inference of population relationships, and show how incomplete sampling and simplified assays can obscure cryptic diversity and bias interpretation. We then examine factors that complicate interpretation of OrNV-based biocontrol outcomes, including diagnostic sensitivity, study design and environmental modulation of host–pathogen interactions. Overall, these examples show that molecular evidence is most informative when marker choice, biological validation and phenotypic data are aligned with the biosecurity question.

## Highlights

• CoxI supports screening but should not be overextended to phenotype.
• Mitogenomic and nuclear data refine invasion-pathway inference.
• Marker-based biosecurity inference requires phenotypic validation.
• OrNV efficacy assessment should include viral-load and trait data.

## 1 Introduction

Invasive pest management is being reshaped by rapid advances in molecular biology and high-resolution genetic technologies. These tools are changing how biosecurity programs detect invasive species, investigate population dynamics, reconstruct invasion pathways and evaluate control strategies (Burgess et al., 2021). The ability to decode genomes at scale has enhanced our understanding of pest evolution, dispersal pathways, resistance mechanisms to pesticides and biocontrol strategies (McGaughran et al., 2023). In biological control, molecular tools can support the discovery, selection and evaluation of pathogens, parasitoids and predators used against invasive pests (Leung et al., 2020)​. By analysing host–pathogen interactions at the genetic level, researchers can better guide the selection and evaluation of biological control agents with improved specificity and efficacy, while minimising potential effects on non-target species (Etebari et al., 2011). However, host resistance evolution, driven by genomic plasticity, remains a major hurdle for sustained control efforts.

In applied biosecurity contexts, molecular evidence increasingly supports early detection, precision diagnostics and improved biocontrol strategies. These technologies can support more targeted surveillance by distinguishing newly introduced populations from established ones, helping biosecurity agencies prioritise high-risk pathways and manage incursion risks more effectively. Additionally, advances in environmental DNA (eDNA) metabarcoding and high-resolution population genetics have improved our ability to track invasion pathways, supporting more effective quarantine and eradication programs (McGaughran et al., 2023).

While the integration of molecular diagnostic tools into pest management and biosecurity has strengthened our ability to detect, track and control invasive species, translating these technologies into robust, field-ready solutions remains challenging (Leung et al., 2020). The complexity of molecular data interpretation, constraints on data quality and reproducibility, and the underrepresentation of many insect taxa in reference datasets remain key barriers, alongside the need to keep molecular tools current as pest populations evolve (Cameron, 2025). Additionally, while these tools offer powerful predictive capabilities, their real-world implementation requires rigorous validation to avoid misinterpretations that could lead to ineffective or even counterproductive management strategies. As we move toward a more genomically informed approach to biosecurity and biocontrol, it is crucial to acknowledge and navigate these challenges to ensure that these innovations translate into sustainable, long-term solutions.

In this article, we use the coconut rhinoceros beetle (CRB), *Oryctes rhinoceros* L. (Coleoptera: Scarabaeidae), as a real-world example of how molecular tools have been applied to an invasive pest. Its recent well documented incursions into new territories in the south Pacific make it an ideal case study to illustrate the complexities of applying and interpreting these new methods effectively. Ongoing research continues to address key questions about the population structure of CRB, the origins of recent incursions, and the genetic relationships among populations involved in new waves of invasion. Additionally, there is ongoing debate regarding the efficacy of Oryctes rhinoceros nudivirus (OrNV) as a biocontrol agent, with concerns over resistance evolution and the variation in virus effectiveness across different CRB populations. Moreover, interpreting OrNV infection in field populations remains challenging because detection outcomes can vary with tissue type, viral load, assay sensitivity and sampling design, complicating comparisons among studies and management contexts. By examining the CRB invasion through a molecular evidence framework, we can better understand the strengths and limitations of these technologies and explore how this data can be integrated with other biosecurity approaches to improve long-term pest control strategies.

## 2 Coconut rhinoceros beetle in the Pacific

### 2.1 Biology, economic importance and management

CRB is a serious threat to livelihoods in the Pacific Islands where coconut palms (*Cocos nucifera*) provide essential resources and coastal protection for five million vulnerable people [see review by Bedford, 2013]. Adult CRB bore into the palm crown to feed, leaving characteristic V-shaped cuts in emerging fronds and, under heavy pressure adult CRB feeding can cause crown collapse and palm death. Over the past two decades, *O. rhinoceros* has invaded several previously pest-free countries and territories, including Guam in 2007, Hawaii in 2013, the Solomon Islands in 2015, and Vanuatu and New Caledonia in 2019 (Etebari et al., 2021b).

Across the Pacific, CRB incursions impose substantial costs, although the dominant impact pathways differ among island systems. In the Solomon Islands, emergency responses were initiated following the 2015 detection, yet CRB became established and projected direct losses to copra and other coconut-derived exports were estimated at approximately US$300 million per year (10–15% of GDP) (Tsatsia et al., 2018). In Hawaii, where palms contribute to urban amenity, tourism value, and high-cost managed landscapes, economic assessments that include palm replacement costs likewise indicate major potential losses (US$3.8 million per year) if CRB spreads broadly across Oahu (Kaufman, 2021).

The economic impact of CRB is not limited to the Pacific Islands. Large areas of the globe are important for palm production and are potentially susceptible to the pest. Climate conditions play a significant role in the distribution and invasion risk of *O. rhinoceros*, with projections indicating that CRB could expand its global range by up to 40% by 2090 (Aidoo et al., 2022; Xu et al., 2022). A recent study shows that suitable areas for the beetle are mainly located between 30°N and 30°S, with highly suitable areas concentrated in northern Oceania, South, East, and Southeast Asia (Fu et al., 2024). Although the study concludes that the pest is unlikely to establish in Australia or New Zealand, it was intercepted 13 times in New Zealand between 2003 and 2023 and 14 times in Australia over a similar period (Hoffmann et al., 2024). These interception rates demonstrate that the pest frequently enters new regions and highlights the ongoing risks to environmentally suitable territories that are currently CRB*-*free, underscoring the importance of strong biosecurity and surveillance in regions where palms are economically significant.

For any invasive pest, early detection enables rapid response and increases the likelihood of containment or eradication, avoiding the long-term costs of ongoing management. In contrast, delayed detection and post-establishment control reduce the chances of success and place substantial financial pressure on newly invaded countries (Hamza et al., 2026). In Hawaii estimates of costs associated with the recent establishment of CRB indicate that mitigation costs can be up to six times higher than those of routine airport monitoring programs (Kaufman, 2021). Molecular and genomic tools can strengthen these efforts by supporting early detection through approaches such as eDNA, pinpointing incursion sources and invasion pathways, distinguishing repeated introductions from local spread, and identifying cryptic population structure that can affect establishment risk. These insights can support more targeted surveillance, better prioritisation of high-risk pathways, and faster, evidence-based response decisions.

Management of CRB has always been challenging, and eradication is usually only realistic when an incursion is detected early and remains localised. In newly detected areas, response programs have included delimiting surveys, movement restrictions, pheromone trapping and removal or treatment of breeding sites. However, once CRB becomes widely established, eradication becomes difficult and management usually shifts toward containment and population suppression. Although removing decaying wood, fallen palms and other organic material that supports larval development is expensive and labour-intensive, sanitation remains one of the most important components of CRB management (Bedford, 1980). Chemical insecticide treatments may be useful in high-value or managed palms and in localised eradication responses, but they are difficult to apply across dispersed smallholder coconut systems. Fungal biocontrol agents such as *Metarhizium anisopliae* have also been investigated, particularly in oil palm systems where breeding sites and trap-based dissemination can be managed more systematically (Moslim et al., 2011).

In the 1960s, OrNV, collected in Malaysia, was successfully introduced into Samoa for CRB control (Marschal, 1970). It was later introduced to other parts of the region, where suppressed CRB populations were also reported, leading to reduced palm damage (Huger, 1966; Bedford, 1986; Huger, 2005). The control of CRB in the South Pacific by OrNV is widely regarded as a landmark example of classical biological control (Caltagirone, 1981). However, since the introduction of OrNV, several reports have indicated possible failures of the biocontrol agent that can manifest as inconsistent OrNV induced mortality, a lack of disease symptoms or low incidence of the virus in wild populations resulting in repeated outbreaks of the beetle (Zelazny et al., 1989).

### 2.2 DNA Barcoding of *O. rhinoceros*

DNA barcoding has played an important role in CRB biosecurity because mitochondrial sequences generally provide a practical and affordable way to confirm species identity and assign specimens to broad maternal lineages. This approach also enabled the development of PCR-RFLP assays that could be applied in settings without advanced sequencing capacity or specialised laboratory facilities. However, while these tools are useful for initial screening and broad haplotype assignment, their results have sometimes been over-interpreted beyond the resolution of the marker.

In 2017, a newly identified mitochondrial haplotype of CRB was suggested to be responsible for recent invasions in the Pacific and to have reduced susceptibility to OrNV (Marshall et al., 2017). Since then, mitochondrial diversity of CRB in the Pacific has generally been discussed in terms of three major cytochrome c oxidase subunit I (*CoxI*)-defined haplotype groups. CRB-S refers mainly to historically established South Pacific populations that were assumed to be susceptible to OrNV, while CRB-G was first identified in Guam-associated populations and later reported from several newly invaded Pacific locations. A third haplotype group, CRB-P, is associated with Papua New Guinea and related regional populations. Here, an individual haplotype refers to a unique *CoxI* sequence, whereas a haplotype group or clade refers to a cluster of closely related haplotypes identified by phylogenetic analysis. Such groups may contain additional sequence variation beyond the diagnostic sites used to assign them. In our regional study, 12 individual haplotypes were assigned to three major haplotype groups (Etebari et al., 2021b).

Subsequent population genetic studies have shown a more complex picture, with multiple mitochondrial lineages present in some countries and evidence of nuclear admixture among populations (Etebari et al., 2021b). For example, Tonga was monotypic for CRB-S, while Fiji contained four CRB-S variants and Samoa contained mainly CRB-S, with one CRB-P variant. The CRB-P group was detected in Papua New Guinea, Vanuatu and parts of Solomon Islands, while CRB-G-associated sequences were detected in Solomon Islands, New Caledonia and the Philippines. Previously published sequences from Guam, Hawaii and Palau also grouped within CRB-G based on partial *CoxI* data. More recently, analysis of 263 CRB specimens identified 72 haplotypes across the 13 mitochondrial protein-coding genes, revealing substantially greater mitochondrial diversity than partial *CoxI* barcoding had resolved (Tay et al., 2025). These patterns show that the CRB-S, CRB-G and CRB-P labels refer to broad mitochondrial lineages that can contain multiple related haplotypes, and that more than one lineage may occur within the same country (Etebari et al., 2021b). Therefore, they should not be treated as discrete biological types or as reliable predictors of invasion origin, damage potential or virus susceptibility.

In this context we critically examine the recent literature and reappraise the current status of CRB in the Pacific region, to better inform biological control programs. Specifically, we focus on the limitations of haplotyping using the mitochondrial *CoxI* gene, highlighting how these constraints affect its application in biosecurity for CRB and how similar interpretive pitfalls can arise in other invasive pest systems where mitochondrial markers are used as proxies for biologically meaningful traits. We also review recent literature on host-virus interactions to investigate whether CRB populations may be developing reduced susceptibility to OrNV.

## 3 Molecular markers for pest haplotyping and invasion-pathway inference

The mitochondrial *CoxI* gene is widely used as a DNA barcode for pest identification and haplotyping because it is easy to amplify from small or degraded specimens, has strong reference library support, and often provides sufficient resolution to distinguish species and delineate broad invasion lineages. The widespread use of *CoxI* should also be understood in historical and practical context. For many non-model insect systems, DNA barcoding was one of the few affordable and widely accessible molecular tools available for species confirmation, preliminary haplotype assignment and broad comparison among populations. It also helped build reference datasets and provided an entry point for many studies where genome-wide data were not yet available. The limitation is therefore not the use of *CoxI* itself, but the extension of single-marker results to questions that require additional nuclear, ecological or phenotypic evidence.

In biosecurity, mitochondrial DNA (mtDNA) sequencing and analyses have proven extremely useful for understanding both historical and recent movements of insects, especially when divergent haplotype groups have independently invaded new regions (Brookes et al., 2020). However, *CoxI* is a single, maternally inherited marker and it does not reliably capture genome-wide gene flow, hybridisation, selection, or traits that matter for management. Consequently, assigning ecological or behavioural meaning to *CoxI* “strains” without supporting nuclear data and phenotypic validation can be misleading. The fall armyworm (*Spodoptera frugiperda*) illustrates these caveats. Mitochondrial haplotypes have often been used to label “maize” and “rice” strains and to infer host preference, but host use in the field does not always match these assignments (Acharya et al., 2021). In some invasive populations, *CoxI* is no longer a reliable indicator of host preference, while nuclear markers such as *Tpi* and genome-wide SNPs reveal admixture or population structure that does not always correspond neatly to mitochondrial strain labels (Schlum et al., 2021). This highlights the risk of treating mitochondrial variation as a reliable proxy for biologically meaningful differences in behaviour or ecology (Nagoshi, 2022). Misinterpreting haplotypes as host-specific “strains” can misdirect surveillance and control towards one crop while the same pest population readily shifts to other hosts, undermining containment efforts and wasting resources. This does not mean that mitochondrial haplotypes can never correspond to biologically meaningful host-associated lineages; rather, such associations need to be demonstrated with independent ecological, behavioural or experimental evidence rather than inferred from the marker alone.

Following the recent CRB invasion in the Pacific Islands, various molecular approaches were employed to identify the different haplotypes of beetles involved (Reil et al., 2016; Marshall et al., 2017; Etebari et al., 2021b). In this review, we use “haplotype group” to refer to a cluster of closely related mitochondrial *CoxI* sequences identified within a particular study, rather than a formally defined taxonomic, biological or phenotypic unit. Because studies have used different sampling schemes, sequence lengths and analytical approaches, these groups are best interpreted within the context of each study rather than treated as fixed labels across the entire CRB invasion history. Reil et al. (2016) investigated mitochondrial and nuclear DNA sequences from CRB collected in Asia and the northern Pacific islands. Based on *CoxI* gene analysis, they identified two major haplotype groups corresponding to two invasion fronts across the region. One haplotype group was found in Guam, Taiwan, Oahu (Hawaii), and Palau, while the other was found in American Samoa, China, Thailand, Vietnam, and Palau. In addition to mitochondrial analysis, they explored the genetic diversity of the nuclear CAD (carbamoyl-phosphate synthetase, aspartate transcarbamoylase, dihydroorotase) gene, which revealed more genetic variation than the mitochondrial *CoxI* gene. However, the authors concluded that the CAD gene lacked the resolution needed to infer invasion pathways (Reil et al., 2016).

Choosing an appropriate molecular marker is critical because *CoxI* often lacks sufficient resolution to distinguish among closely related populations. The CRB-G haplotype should not be treated as a discrete entity, as closely related *CoxI* haplotypes occur across the native range, including the Philippines, Taiwan, and Indonesia (Marshall et al., 2017). Consistent with this, Tay et al. (2024) analysed complete mitochondrial genomes from beetles sampled in Guam and other locations. They showed that Guam specimens shared an identical mitogenome across all 13 protein-coding genes, forming a distinct mitochondrial lineage that differed from specimens collected elsewhere, including ‘non-Guam’ beetles previously grouped as CRB-G based on partial *CoxI* sequences. This mitogenome-level separation indicates that the beetles invading other Pacific locations that were assessed in their study were unlikely to have originated from Guam (Tay et al., 2024). It is more likely that the first beetles that invaded Guam originated from Asia, but there is currently insufficient data to confidently identify the precise origin of the beetles. Therefore, the group of beetles referred to as CRB-G should not be treated as a single, well-defined biological entity. There is also no evidence that all beetles assigned to this *CoxI*-defined group are inherently more destructive than other CRB populations. Another recent study using whole mitochondrial genome sequencing proposed that CRB populations in Japan, Palau, Guam, Rota and Hawaii were most likely introduced from the Philippines or Taiwan (Tay et al., 2025).

In many CRB-affected countries, biosecurity agencies lack the infrastructure for molecular identification and haplotyping and instead rely on overseas laboratories. Despite this, substantial effort has been directed towards mapping regional haplotypes, with partial mitochondrial *CoxI* sequences used to identify variable sites that might distinguish the putatively OrNV-susceptible CRB-S haplotype from the putatively resistant CRB-G population. However, no functional link has been demonstrated between *CoxI* variation and OrNV susceptibility in CRB. Therefore, using this genetic region alone to infer OrNV resistant or susceptible CRB populations is not well supported (see Section 5).

Moreover, a single nucleotide polymorphism (SNP) at position 288 within a 676 bp *CoxI* fragment was used to develop a PCR-RFLP assay to screen putative OrNV-susceptible and resistant CRB populations (Marshall et al., 2017).

**Fig. 1:**
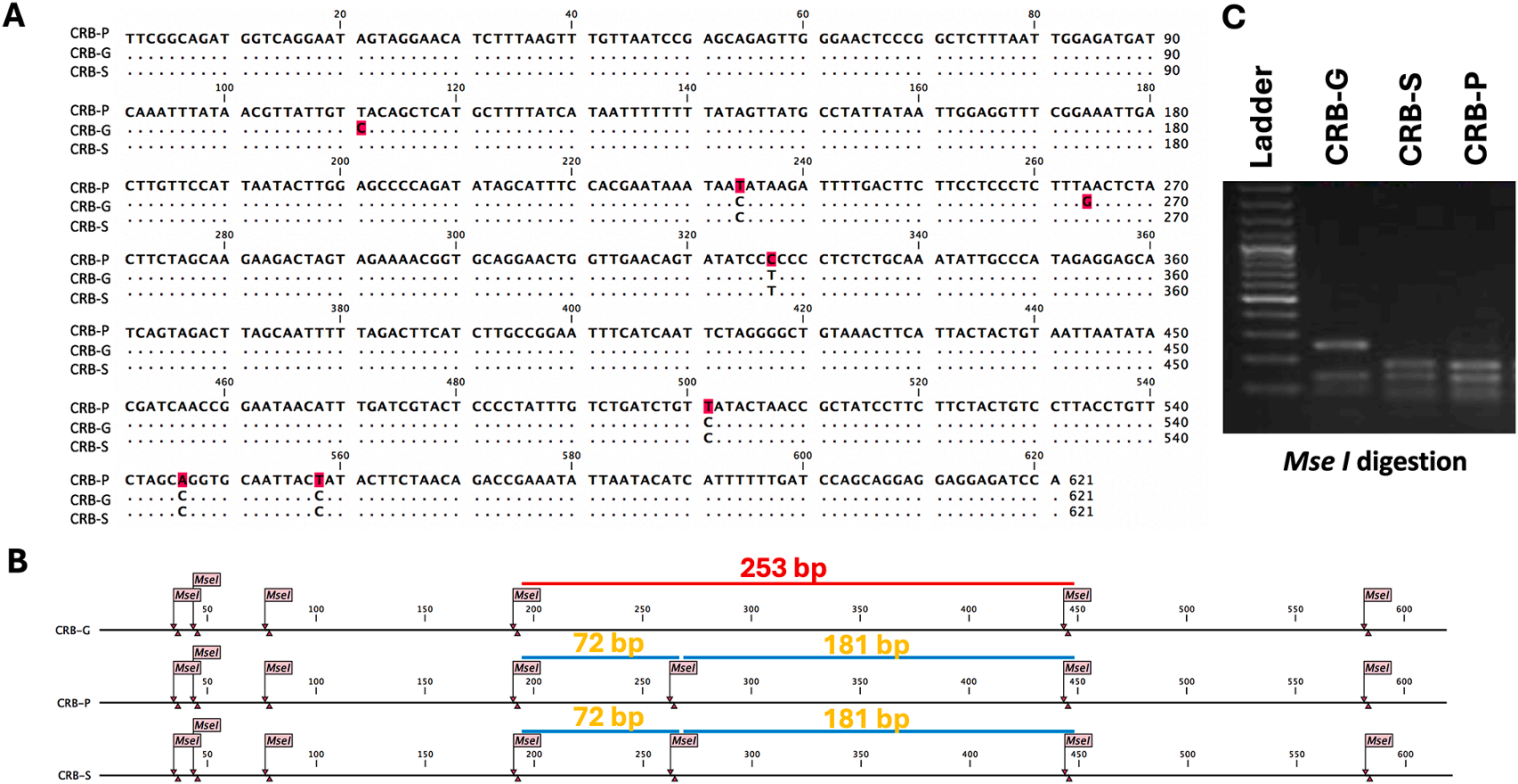
Limited resolution of the *MseI* based PCR-RFLP assay for mitochondrial *CoxI* haplotyping in *O. rhinoceros*. (A) Alignment of the *CoxI* amplicon shows that multiple polymorphic sites occur across CRB lineages, but the diagnostic PCR-RFLP relies on a single *MseI* (TTAA) restriction site. (B) Predicted restriction profiles indicate that *MseI* digestion separates CRB-G from the other lineages (253 bp vs 181 bp plus 72 bp), but yields the same fragment pattern for CRB-P and CRB-S. (C) Representative gel confirms indistinguishable banding between CRB-P and CRB-S, demonstrating that this assay can oversimplify regional diversity and cannot reliably discriminate S from P populations.

However, because the assay relies on a single *MseI* restriction site, it separates CRB-G from other lineages but does not distinguish CRB-S from CRB-P. It can therefore oversimplify CRB genetic diversity and miss additional mitochondrial lineages already present in the region, including the Papua New Guinea-associated haplotype group referred to as CRB-P (Fig. 1). Despite these limitations, the assay has been widely applied to map *CoxI*-defined haplotypes across the Pacific and to support biosecurity programs aimed at limiting the movement of newly detected lineages.

Although an earlier report based on partial *CoxI* gene sequences identified CRB-G as the invasive haplotype in the Solomon Islands, comprehensive studies using genotyping by sequencing and mitogenome analysis confirmed the presence of more than one mitochondrial haplotype in the country (Etebari et al., 2021b; Filipovic et al., 2021). This work suggested the possibility of several invasion fronts in the Solomon Islands: one from neighbouring Papua New Guinea, another potentially originating from Southeast Asia, though beetles may have arrived in Solomon Islands through intermediary countries, and another from populations previously established in other South Pacific island countries.

The identification of a CRB-G *CoxI* haplotype in any country does not imply that these beetles originated from Guam or that they share the same wider genomic background as the CRB population established in Guam.

Despite the presence of major *CoxI* haplotypes in the Solomon Islands, population genetic analysis of nuclear DNA data showed that the CRB population there was well-mixed genetically. The CRB population on Guadalcanal, the country's most populous island, with its largest port and where CRB was first detected, was closely related to the CRB population in Papua New Guinea (Etebari et al., 2021b). When divergent mitochondrial haplotypes of a species overlap and interbreed, the resulting genetic admixture and gene flow between haplotypes means that mitochondrial genome information is no longer a reliable marker for biological differences that may have been detected when they were in isolation (Toon et al., 2016; Brookes et al., 2020).

Recently, the genome sequence of CRB became available (Filipović et al., 2022). Preliminary analysis using the full genome sequence and other publicly available deep-sequencing data indicated that CRB populations from Guam and Hawaii are closely related to each other, but genetically distinct from individuals collected in the Solomon Islands, even though partial *CoxI* data had placed these populations within closely related mitochondrial haplotype groups (Filipović, 2023). This new evidence suggests that the beetles in the Solomon Islands and Guam are unlikely to share the same origin or invasion path. Further support for this is provided by recent analyses of the whole mitogenome, as well as phylogenetic analysis using partial *APT6* (446 bp) and partial *COIII* (422 bp) gene sequences, which indicates that the population in Guam is not the source of the current invasions of CRB-G in other countries (Tay et al., 2024).

This body of research indicates that the current *CoxI*-based haplotype diagnostics are unlikely to reflect any significant biological differences between CRB populations in the Pacific. As previously described in places such as the Solomon Islands, Palau, and Papua New Guinea, where multiple haplotype groups are present and genetic admixture occurs between them, the utility of *CoxI*-based diagnostics for tracking the movement of any given haplotype is limited, as beetles can only be traced through the maternal line (Reil et al., 2018; Etebari et al., 2021b). For example, male beetles from the CRB-G *CoxI* haplotype that mate with females from a different haplotype would produce offspring undetectable as CRB-G using *CoxI* diagnostics, even though they would carry paternal nuclear genes, which may include those mediating interactions with viruses and other pathogens. Similarly, females from the CRB-G haplotype group that mate with males from another haplotype would produce offspring detectable as belonging to the CRB-G *CoxI* haplotype, but their nuclear genome would be a mix of both parents.

A recent study suggests using an alternative mtDNA marker for distinguishing between Guam and non-Guam CRB-G lineages (Tay et al., 2024). While the identification of this new marker would enhance our monitoring capabilities and improve understanding of the incursion pathways for this pest, there is still insufficient evidence to link any mitochondrial haplotype of CRB to biological traits, such as resistance to viruses or increased invasiveness, in either Guam or non-Guam populations of these beetles. Although using additional mitochondrial genes as alternative barcode markers, such as ATP6 (a mitochondrial protein-coding gene), may help resolve some complexities in tracing the origin of the Guam population, the broader limitations of mitochondrial barcoding remain. For example, reliance solely on ATP6 can lead to misidentification, with *O. nasicornis* potentially being misclassified as *O. rhinoceros* (Tay et al., 2024).

## 4 Beyond partial *CoxI*: higher-resolution approaches for population and invasion inference

Identification of the source of an invasion and detecting multiple invasion fronts can be challenging using partial mitochondrial *CoxI*, especially when several *CoxI* haplotypes are present in nearby geographical regions, or when there is insufficient variation in their sequences. CRB invasions of Pacific islands are likely human-mediated, consistent with its status as a “hitchhiker” pest, and they are probably driven by increasing trade and interconnectivity between islands, which can facilitate the movement of beetles and associated genetic diversity (Reil et al., 2016; Etebari et al., 2021b). The incursions of CRB into New Caledonia and Vanuatu in 2019 involved two different CRB haplotypes, CRB-G and CRB-P respectively, suggesting that the ability of beetles to expand their range is independent of their mitochondrial haplotype group.

**Fig. 2:**
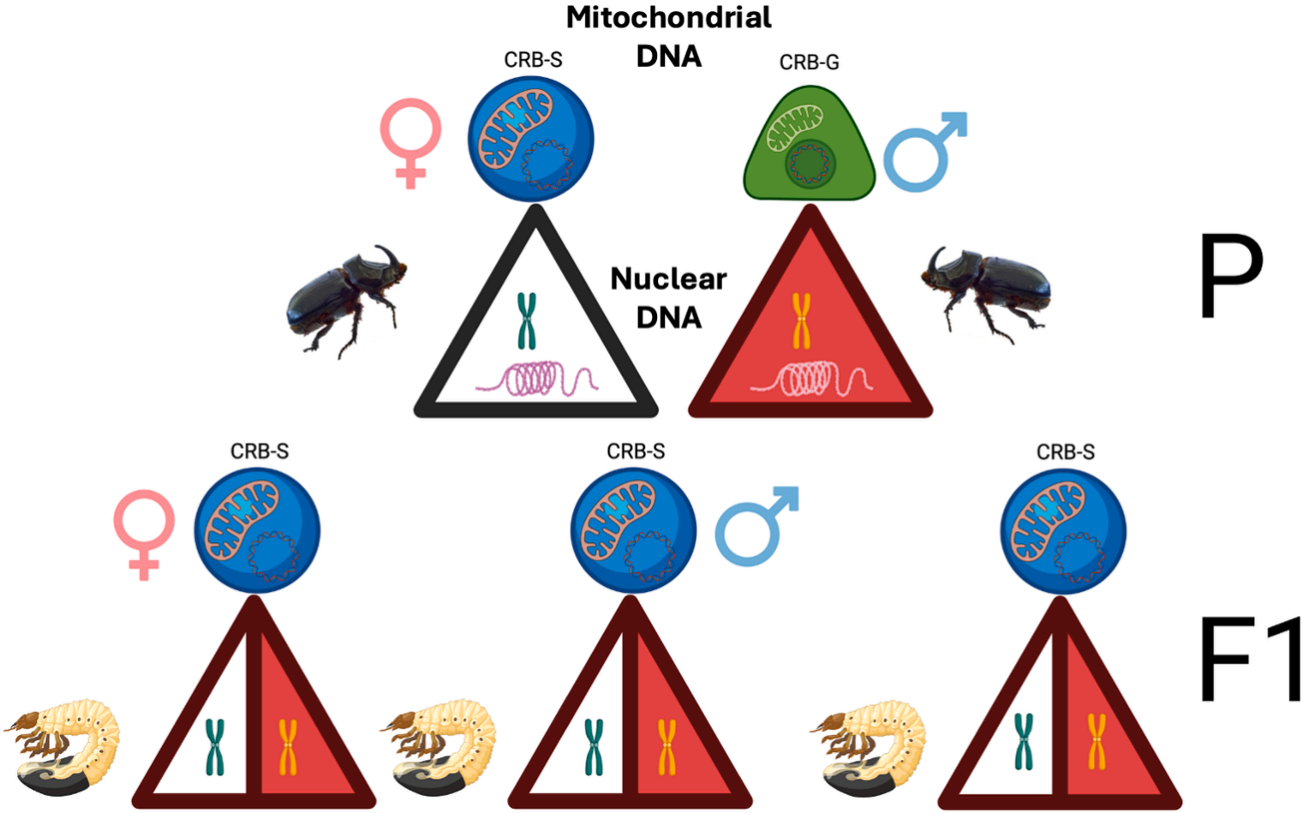
Conceptual illustration of mitochondrial–nuclear discordance and why *CoxI* haplotypes may not predict phenotype. Adult beetles are shown with mitochondrial DNA (maternal inheritance; blue for CRB-S, green for CRB-G) and nuclear DNA (biparental inheritance; red/black triangles). Following mating between individuals from different lineages, F1 offspring inherit the mother’s mitochondrial haplotype, while their nuclear genome is a recombined mixture of both parents. As a result, individuals can carry the same *CoxI* haplotype yet differ in nuclear background and associated traits, which can confound attempts to link *CoxI*-based “haplotypes” to complex phenotypes such as virus susceptibility or behaviour.

Further, although the CRB-P haplotype detected in Vanuatu is also the more abundant haplotype in Papua New Guinea, this does not necessarily mean that the Vanuatu population originated directly from Papua New Guinea (Etebari et al., 2021b). For example, beetles assigned to CRB-P by mitochondrial markers could be the progeny of a CRB-P female and a male from another haplotype group, such as CRB-G, in a location where both haplotypes coexist, such as the Solomon Islands (Fig. 2).

A recent study analysed draft mitogenomes from 263 historical and contemporary CRB specimens collected across its native and introduced ranges (Tay et al., 2025). The authors combined all 13 mitochondrial protein-coding genes, comprising approximately 11.1 kb, and used phylogenetic analysis, haplotype networks, and population-differentiation estimates to compare maternal lineages. This approach identified 72 mitogenome haplotypes, revealing substantially greater diversity than partial *CoxI* sequencing. The analysis did not support Guam as a bridgehead for subsequent Pacific invasions and indicated that CRB collected in Guam and Hawaii had separate maternal origins. The mitogenome data also identified maternal affinities between beetles collected in Papua New Guinea and Solomon Islands and Malaysian samples while insects from Samoa and Fiji showed maternal affinities with Sri Lankan samples. A second mitogenome lineage was also detected among recently collected Guam specimens, suggesting an additional introduction that was not resolved using partial *CoxI*. The authors concluded that whole-mitogenome data can substantially improve maternal source attribution and distinguish invasion events obscured by partial barcoding. However, because mitogenomes trace only maternal inheritance, they recommended combining these data with nuclear population-genomic analyses and broader sampling of native and invaded populations (Tay et al., 2025) as has been suggested previously (Etebari et al., 2021b).

Several studies on other insects have shown that monitoring genetic variation in both the host and the biocontrol agent is crucial for maintaining the success of biological control programs. This requires the use of appropriate molecular markers or high-resolution genotyping techniques. Using an unsuitable marker or method can mislead the biological control strategy. For instance, biological control of the invasive Argentine stem weevil, *Listronotus bonariensis* (Kuschel) (Coleoptera: Curculionidae) by the parasitoid *Microctonus hyperodae* Loan (Hymenoptera: Braconidae) was initiated in New Zealand pastures in the 1990s (Harrop et al., 2020). Within three years of its initial release, the parasitoid had established and effectively controlled the pest but the biological control efficacy declined after about 14 generation (Goldson and Tomasetto, 2016). Early studies using randomly amplified polymorphic DNA (RAPD) markers and mitochondrial *CoxI* sequencing suggested a high degree of genetic similarity and identified only a single *CoxI* haplotype in the pest population (Williams et al., 1994). In contrast, a recent study using a genotyping-by-sequencing (GBS) approach revealed significant genetic diversity within and between host populations and it has been suggested that this high level of heterozygosity in the Argentine stem weevil population enabled it to evolve resistance to the biological control agent (Harrop et al., 2020). The relevance to CRB is that changes in biocontrol performance cannot be attributed confidently to mitochondrial lineages without investigating nuclear diversity in the host and genetic variation in the biological control agent.

This does not mean that RAPD markers or *CoxI* sequencing were inappropriate at the time. Rather, these methods reflected the tools that were available, affordable and practical for many non-model insect systems. Their use provided an important foundation for later studies, but newer approaches such as GBS and whole-genome sequencing now allow earlier conclusions about population structure, invasion pathways and adaptive variation to be tested with much greater resolution.

The value of complementing mitochondrial markers with nuclear data is also illustrated by studies of other invasive pests. Recently, Wongnikong et al. (2021) explored the invasion history of *Bemisia argentifolii* into Australia by analysing its *CoxI* gene haplotype network and developing microsatellite loci based on whole-genome sequencing (Wongnikong et al., 2021). Australian *B. argentifolii* mostly share the same *CoxI* haplotype as invasive populations established elsewhere around the world (Wongnikong et al., 2021) but microsatellite data based on whole-genome sequencing revealed a high level of gene flow across populations and confirmed several invasions into Australia. This species is a well-known vector of plant viruses and rapidly develops resistance to insecticides (Horowitz, 2020). Currently, the Australian population is free from these undesirable traits, but any new incursion, even from the same *CoxI* haplotype groups, could introduce these traits, making them difficult to identify and contain (Wongnikong et al., 2021). This study highlights the importance of preventing or minimising pest movement, even when the species is already established, through continued quarantine measures and biosecurity practices, which are critical for successful pest control programs. This provides a direct lesson for CRB biosecurity, even where the pest and a particular *CoxI* lineage are already established, subsequent incursions may introduce nuclear genetic variation affecting invasiveness or susceptibility to control measures.

Currently, CRB is well established in many Pacific countries due to historical introductions, but these nations cannot relax biosecurity or quarantine measures targeting the pest. Each new incursion can introduce new genetic material, potentially leading to increased adaptation to existing management programs and environmental factors. Previous genotyping by sequencing has clearly demonstrated the complexity of genetic material in CRB in Samoa, the first country in the Pacific to be invaded by CRB in the early 1900s (Etebari et al., 2021b). Higher levels of genetic admixture were detected on the island of Upolu, where the main port is located, than on the less directly exposed island of Savaii. Although human-assisted transport drives new incursions, the nuclear genomic data suggested limited subsequent gene flow among the established populations examined in Fiji, Samoa and Tonga, each of which remained genetically distinct (Etebari et al., 2021b).

When host mitochondrial markers provide limited resolution, associated microorganisms or viruses may offer complementary evidence for tracing invasion pathways (Tabein et al., 2025). For CRB, this possibility is illustrated by the discovery of Oryctes rhinoceros picorna-like virus 1 (OrPV1) in larvae from Taiwan (Etebari et al., 2020b). This virus was highly prevalent in the Taiwan population but was not detected in CRB populations sampled from the South Pacific islands or the Philippines, suggesting that population-associated viruses could provide useful supplementary markers if validated with broader sampling. Similar approaches have been informative in other invasive insects; for example, in *Aedes aegypti* from Madeira, Portugal, viral sequence data and nuclear *kdr* allele frequencies helped refine invasion-source inference when mitochondrial markers alone had limited resolution (Seixas et al., 2013). For CRB, metagenomic and virome-based approaches should therefore be viewed as complementary tools, not replacements for host genomic data, that may help distinguish invasion sources where *CoxI* variation is insufficient.

## 5 Linking a *CoxI* haplotype to phenotype or complex traits

Linking the mitochondrial genotype to phenotype is a complex task, raising several issues due to the unique characteristics of mitochondria, such as their non-Mendelian inheritance, and numerous unknowns in gene expression and post-transcriptional, and post-translational modifications (Ghiselli and Milani, 2020). Each single-nucleotide variant (SNV) on mtDNA must either directly affect mitochondrial function or be in linkage disequilibrium with variants known to influence mitochondrial metabolism (Elson et al., 2007). These important principles must be considered to avoid misinterpretations when linking mitochondrial genotype to phenotype.

The CRB case provides a useful example of how phenotype can be incorrectly attributed to a *CoxI*-defined haplotype group. Anecdotal reports have sometimes attributed distinctive biological or behavioural traits to CRB-G without sufficient experimental evidence. One example is the claim that CRB-G is poorly responsive to pheromone traps baited with commercial lures, which has been cited as a constraint for surveillance in newly invaded countries. However, a detailed chemical analysis found no qualitative or quantitative differences in aggregation pheromone composition between the two major mitochondrial groups established in the region, CRB-S and CRB-G; both produced ethyl (R)-4-methyloctanoate (Hall et al., 2022). This example illustrates why *CoxI*-defined groups should not be treated as proxies for behavioural traits unless the association is supported by independent phenotypic evidence.

Responses to virus infection, susceptibility to biological control agents, host preference, fitness and environmental adaptation are complex traits that are unlikely to be explained by variation in a single mitochondrial barcode marker alone. Although mitochondrial variation can sometimes be associated with phenotypic differences, no functional link has been demonstrated between *CoxI* variation and OrNV susceptibility in CRB. For example, a previous study demonstrated that CRB from the Philippines and the Solomon Islands exhibited distinct immune responses to OrNV infection, despite belonging to the same mitochondrial lineage (Etebari et al., 2021a). A comparative analysis of infected and non-infected CRB revealed that specific gene sets were induced by viral infection, with a much stronger response observed in beetles from the Solomon Islands, where the pest had recently arrived, than in beetles from the Philippines, where the pest is native, or those from Fiji and Papua New Guinea, where the pest is has long been established (Etebari et al., 2021a). In addition to basic genetics, these country-specific immune responses provide additional evidence that mitochondrial markers, such as *CoxI*, are not appropriate indicators for traits like virus resistance. Thus, great care must be exercised if mitochondrial genotype is used to describe a phenotype, especially when the phenotype is not regulated by mtDNA or when the mechanisms controlling the phenotype are not fully associated with common mtDNA variants. In some instances, notably in cases of arthropod resistance to pesticides, mtDNA genotypes can be used to distinguish phenotypes (Van Leeuwen et al., 2008; Seixas et al., 2013; Ding et al., 2020). Typically, mitochondrial genes do not directly confer pesticide resistance but in red spider mites, where resistance is linked to mutations in cytochrome b (*cytb*), a mitochondrially encoded protein in the respiratory pathway (Van Leeuwen et al., 2008) and nonsynonymous mutations in the *cytb* gene contribute to resistance. As such, *cytb* sequencing can be used for haplotyping resistant individuals, but it remains a rare example of non-Mendelian inheritance affecting phenotype, in this case pesticide resistance.

Returning to CRB, putative resistance to OrNV has been attributed to single nucleotide variants in the mitochondrial *CoxI* gene, with this haplotype described as a “biotype” (Marshall et al., 2017). As noted earlier, there is no convincing evidence for this association, it is unsupported by the biological function of the marker and lacks phenotypic validation. In the following sections we consider the potential resistance of CRB to OrNV, discussing the genetic and phenotypic factors that might underpin it.

## 6 The biological control agent, Oryctes rhinoceros nudivirus (OrNV)

The first attempts to introduce OrNV (previously known as *Rhabdionvirus*) into populations of CRB began in Western Samoa in 1967, where no viral disease had previously been detected (Marschal, 1970). Several years after introduction, OrNV incidence in wild CRB reached 30 to 50%, yet beetle abundance and palm damage increased during this period. Re-release of the virus into the already infected population was shown to raise infection levels further and reduce beetle numbers and damage (Marschall and Ioane, 1982). Rather than rapid mortality, OrNV induces a chronic infection in adult hosts that does not necessarily cause immediate population declines (Zelazny, 1977b, a) and introduction of the virus into new islands sometimes reduces adult populations by only 10-20% of their original density (Zelazny and Alfiler, 1991). For example in Fiji CRB damage to palms decreased significantly 12-18 months after OrNV establishment but CRB was never eradicated, and some level of host plant damage persisted (Bedford, 1986). Further, OrNV is most effective when CRB population density is low. CRB lays eggs in rotting wood and other decaying organic material, where larvae subsequently develop. When large numbers of breeding sites are created, for example after cyclone damage to palms, CRB populations can surge and outbreaks may occur despite the presence of the virus (Bedford, 1986).

Several different approaches have been used to examine OrNV infection rates in CRB populations, leading to widely varying estimates of virus incidence (Gopal et al., 2002; Moslim et al., 2011; Marshall et al., 2017; Rahayuwati et al., 2020; Etebari et al., 2021b). Assessing visible symptoms of the disease, including by histological methods, in larvae or adult insects can be an unreliable method to estimate virus incidence or infection rate in a population as asymptomatic individuals can lead to underestimates. Many previous studies that have used methods other than standard PCR tests to detect OrNV have reported morbidity rates rather than actual virus incidence. For example, based on histopathological observations only 5% of 6,627 larvae and 22% of 307 *O. rhinoceros* adults examined showed signs of viral disease (Gopal et al., 2002). In contrast in another study, PCR diagnoses have indicated that virus incidence in adults caught in traps could be greater than 98% (Moslim et al., 2011). Estimates of disease incidence need to be interpreted with caution as they can be significantly affected by the methods and techniques used for identification, the stage of insect that is investigated and the environment in which samples were collected.

In surveys conducted in 2019-2020, a high incidence of OrNV in adult beetles across the South Pacific islands was detected, with infection rates ranging from 63% to 95% in all haplotype groups where 10 or more beetles were tested (Etebari et al., 2021b). However, a high incidence of virus in the population does not necessarily mean that symptoms of the disease will be evident in all infected beetles. Marshall et al. (2017) reported that the natural infection rate of OrNV ranged from 45% to 65% in CRB populations from several countries in the region. The differences between estimates in the two studies are likely due to the use of different tissues for diagnostics or working with overly diluted DNA. This highlights a fundamental methodological issue: diagnostic sensitivity can change substantially depending on the tissue selected for extraction and the degree of DNA dilution, either can reduce viral template below the detection threshold and lead to underestimation of OrNV prevalence.

Overall, different studies consistently show a high incidence of OrNV in CRB field populations that is accompanied by a low morbidity rate, raising questions about the capacity of the virus to control the pest. There is significant genetic variation between geographically distinct CRB populations (Reil et al., 2018; Etebari et al., 2021b) and since the introduction of the virus co-evolution between the host and pathogen is likely. Such interactions can maintain both resistant and susceptible alleles within a host population through negative frequency-dependent selection (Duxbury et al., 2019). The ability of a biological control agent to co-evolve with its host is critical for the long-term success of a biological control program and many host-pathogen or host-parasitoid interactions studies have demonstrated that genetic variation between the host and control agent can lead to rapid evolution of resistance in the host (Duxbury et al., 2019; Harrop et al., 2020). For example, a large cross-infection experiment involving four *Drosophila* species and different host-specific isolates of sigma virus suggested that host-pathogen co-evolution increases genetic variation in susceptibility to infection, which is regulated by the presence of major-effect resistance polymorphisms within populations (Duxbury et al., 2019). More broadly, translating knowledge of insect-virus interactions into effective biocontrol requires experimental confirmation of infectivity, host specificity and field efficacy, rather than sequence detection alone (Nolan and Etebari, 2025).

## 7 Difficulties in interpreting efficacy of OrNV and susceptibility of different CRB populations

Claims of “haplotype resistance” often arise when control outcomes are poor and virus detection in field beetles is low. Following the unsuccessful OrNV release in Guam in 2007, resistance was proposed and linked to the CRB-G haplotype (Marshall et al., 2017). However, absence of detectable infection in field-caught beetles is not, on its own, evidence of resistance. Subsequent studies recovered full OrNV genomes from CRB-G associated populations (for example in Malaysia, Palau, and the Solomon Islands), demonstrating that CRB-G can be infected by the pathogen and that the earlier inference was likely confounded by sampling, detection sensitivity, infection dynamics, or local ecological conditions (Etebari et al., 2020a; Tanaka et al., 2021; Anggraini et al., 2023). Robust evidence for host resistance requires comparative bioassays across CRB populations and OrNV isolates, with appropriate controls, including a reference population with known susceptibility and confirmation of infection success.

A practical way to strengthen inference is to integrate viral load measurement into bioassays. Background infection in “control” beetles is common when they are collected from the field (Etebari et al., 2021b) and studies that report mortality outcomes without confirming post-challenge infection (for example, via RT-qPCR viral load) and without reporting baseline infection prevalence in the control group are difficult to interpret. Apparent ‘no effect’ may actually reflect failed exposure, low replication, or pre-existing virus infection rather than resistance to the pathogen [for example, (Barrera et al., 2021)].

This approach mirrors best practice in other insect-virus systems, where resistance alleles are supported by consistently lower viral loads post-infection rather than mortality alone (Duxbury et al., 2019; Magwire et al., 2012). Under standard challenge conditions, consistently lower viral loads in a CRB population compared with those in a known susceptible population could support a hypothesis of resistance. This method has been employed in *Drosophila melanogaster* Sigma virus (DMelSV) host-pathogen studies where two genes (*p62* and *CHKov*) confer protection against infection. In these studies, viral loads 15 days post-infection showed a significant reduction in lines carrying both resistance alleles compared to susceptible populations with at least one susceptible allele (Magwire et al., 2012; Duxbury et al., 2019).

Finally, focusing solely on adult mortality can underestimate OrNV impact. Nudiviruses often produce chronic infections with sublethal effects on feeding, flight, and reproduction, and infected adults may show few external signs of disease (Zelazny, 1977b; Burand, 1998; Unckless, 2011). Therefore, mortality is not necessary an appropriate metric in bioassays intended to compare susceptibility of CRB populations or to assess the performance of isolates. Rather, these studies should incorporate measurements of relevant responses to infection and disease such as fecundity, behaviour, and flight capacity and which have been linked to population suppression.

Another common source of misinterpretation is attributing poor field outcomes to host “resistance” when they may instead reflect how the virus was produced and the history of the isolate used. OrNV has been propagated for decades in the DSIR-HA-1179 cell line derived from *Heteronychus arator* (Crawford, 1981; Crawford, 1982). However, this culture is slow-growing and technically limiting for scaled production, and it often requires serum for optimal growth (Pushparajan et al., 2013; Reid et al., 2016). Long-term amplification in a non-target or facultative host can select variants that perform well *in vitro* but differ in yield, stability, or virulence in CRB. This matters because an attenuated or poorly matched production-derived isolate can produce low infection success or result in weak population suppression that may be misread as resistance, reinforcing the need to pair efficacy trials with infection confirmation and viral load quantification. To mitigate these risks, the establishment of a new cell line derived from CRB has been recommended for OrNV production (Bedford, 2018; Nolan and Etebari, 2025).

**Fig. 3:**
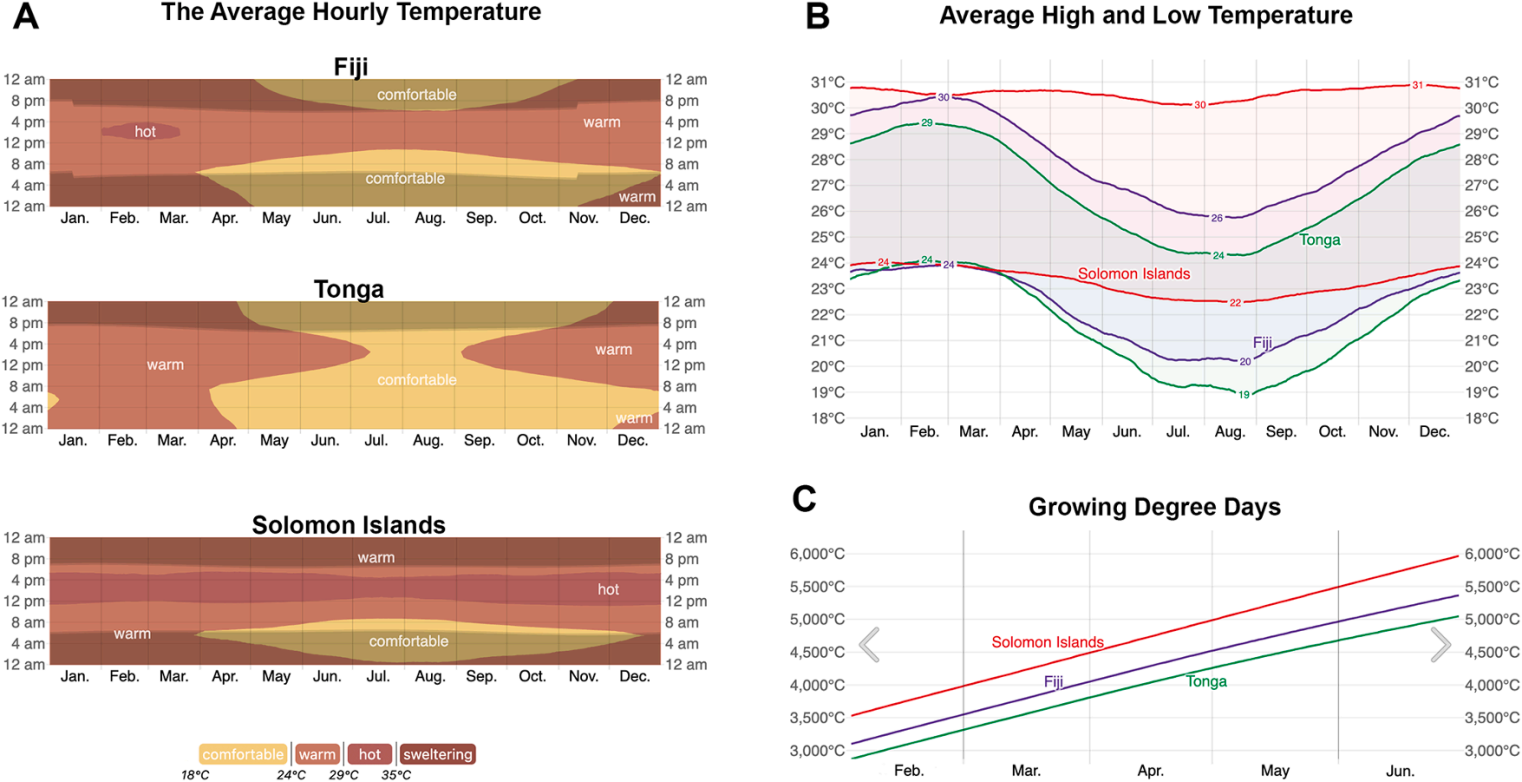
Descriptive comparison of annual temperature profiles for selected South Pacific locations where CRB and OrNV have been reported. Fiji and Tonga represent locations where OrNV has historically contributed to CRB suppression, whereas OrNV has been reported to provide limited control in the Solomon Islands following recent CRB establishment. The figure is intended to illustrate regional climatic differences only and should be interpreted as hypothesis-generating rather than evidence of a causal relationship between temperature and OrNV efficacy. (A) Average hourly temperature, colour-coded into temperature bands; shaded overlays indicate night and civil twilight. (B) Daily average high and low air temperature at 2 m above ground. (C) Accumulated growing degree days over the year, calculated using a base temperature of 10°C. Climate data source: http://weatherspark.com.

Environmental conditions, particularly temperature, may contribute to regional variation in OrNV infection outcomes, but this remains poorly tested in CRB. Temperature can influence insect physiology, immune responses and viral replication, and may therefore affect host–pathogen interactions (Dalmon et al., 2019; Bernardo-Cravo et al., 2020; Tesla et al., 2022; Ferguson and Adamo, 2023; Wang et al., 2023). This is supported by recent work in another coleopteran system, where temperature altered lesser mealworm (*Alphitobius diaperinus*) susceptibility to *Beauveria bassiana*, with moderate heat reducing susceptibility and more extreme heat producing different host–pathogen outcomes (Rice and Furlong, 2026). In Solomon Islands mean diurnal temperatures are high and vary little throughout the year (Fig. 3), such conditions might account for the different responses of CRB to OrNV infection there compared with Fiji and Tonga, where temperatures are cooler but less stable (Fig. 3).

A previous study showed that adult beetles from the Solomon Islands had elevated expression of several immune-related genes, including *coleoptericin, cecropin*-like genes and serpin, following persistent OrNV infection (Etebari et al., 2021a). Temperature-dependent expression of immune-related genes has also been reported in other insects, including *Plutella xylostella* (Wang et al., 2023). However, whether temperature contributes to these responses in CRB, or alters OrNV replication and field efficacy, remains unknown. Controlled experiments are therefore needed to compare viral loads, infection outcomes and host immune responses across defined temperature regimes using standardised CRB populations and OrNV isolates.

## 8 Conclusion and practical recommendations

Molecular tools are reshaping invasive-pest biosecurity, but their value depends on matching the evidence to the management question. *CoxI* barcoding remains a valuable, accessible and cost-effective tool for species identification, initial screening and broad maternal-lineage assignment, particularly when genome-wide approaches are unavailable. It has also supported the development of reference datasets and provided a foundation for subsequent higher-resolution studies. Earlier barcoding research should therefore be understood within its technological and practical context. The CRB case demonstrates that limitations arise not from using *CoxI*, but from extending its interpretation to invasion sources, population structure, phenotypes or biocontrol performance without supporting evidence.

A more reliable path forward is to treat mitochondrial markers as an entry point, not an endpoint. For source attribution and population-level questions, mitochondrial markers should be complemented by nuclear genomic data and representative sampling across native and invaded ranges. Temporal sampling can help distinguish new introductions from local spread. Specimens should also be linked to voucher material with standardised metadata. Proposed associations between molecular markers and traits such as propensity to cause damage, pheromone response or virus susceptibility require controlled phenotypic validation. Assessment of OrNV efficacy cannot rely solely on the presence or absence of detectable virus. Appropriate, sensitive molecular diagnostic assays are needed to confirm infection and quantify viral load, together with suitable susceptible reference populations. Mortality should be considered alongside sublethal effects, while variation among host populations, virus isolates and environmental conditions should be incorporated into experimental designs.

The broader lesson is transferable to other invasive pests where “strains” are inferred from mitochondrial variation without consistent ecological validation. Biosecurity decisions are most defensible when genomic signals are interpreted within a framework that tests assumptions, quantifies uncertainty, and links markers to mechanisms. Applying that discipline will improve the accuracy of source attribution, the targeting of high-risk pathways, and the design of durable biocontrol strategies, reducing the likelihood that genomic tools amplify noise rather than strengthen action.

## Declaration of competing interest

The authors declare that they have no known competing financial interests or personal relationships that could have appeared to influence the work reported in this paper.

## Data Availability

This review did not generate new datasets. All information analysed is from published sources cited in the reference list.
